# Identification of differentially methylated regions (DMRs) of neuronatin in mice

**DOI:** 10.1186/s40064-016-3721-0

**Published:** 2016-11-28

**Authors:** Yuxin Xu, Zhiquan Liu, Tiedong Wang, Xianju Chen, Jichao Deng, Mao Chen, Zhanjun Li

**Affiliations:** College of Animal Science, Jilin University, 5333#, Xi’an Road, Changchun, 130062 China

**Keywords:** *NNAT*, DMR, Mice, Isoform

## Abstract

**Background:**

Neuronatin (*NNAT*) is a paternal-inherited imprinted gene, first discovered in the rat neonatal brain, where it plays vital roles for neuronal growth, brain development, and metabolic regulation. The maternal imprint of *NNAT* has been identified in mice; however, the differentially methylated regions (DMRs) involved in the monoallelic expression of *NNAT* have not yet been investigated.

**Results:**

In this study, we confirmed expression of two isoforms of the *NNAT* (α and β) in the mice brain via quantitative RT-PCR. Additionally, the methylation profile of the CpG island located in the *NNAT* gene locus was determined in the mice liver, brain, sperm, and the MII oocyte via bisulfite sequencing PCR.

**Conclusion:**

In summary, we provide the first evidence for tissue- and gamete-specific methylation patterns of CpG3 that are located on exon 1, to be putative DMR of *NNAT* in mice.

## Background

Genomic imprinting leads to unequal expression of paternal and maternal alleles in offspring, which is essential for normal embryogenesis, fetal growth, and functional postnatal behavior (Surani 1998; Li et al. 1999; Das et al. 2013). The differentially methylated regions (DMRs) are established during gametogenesis and regulate the parent-specific expression of imprinted genes. DMRs with allele specific methylation have also been used in cancer diagnosis (Ushijima 2005; Bonin et al. 2016). Although 125 imprinted genes have been identified in mice to date (according to the Gene imprint database; http://www.geneimprint.com/), very little has been published about the DMRs of those imprinted genes (Gu et al. 2014).

*NNAT* is a highly conserved imprinted gene among humans, mice, cattle, and pigs, containing two alternatively spliced transcripts (α and β) (Cheng et al. 2007; Schulz et al. 2009). Previous studies have revealed the parental-specific expression of *NNAT* to be associated with the methylation status of the CpG island located in the *NNAT* promoter sequence of pigs (Chen et al. 2014; Gu et al. 2014) and rabbits (Duan et al. 2015). A second intronic DNA sequence within the mouse *NNAT* with a length of 250 bp was defined as putative DMR and was revealed to act as a transcriptional activator in Drosophila (Sowpati et al. 2008). In addition, the hyper methylation of human *NNAT* frequently occurs in pediatric acute leukemia and Wilms Tumors (Kuerbitz et al. 2002; Hubertus et al. 2013).

All of these previously published findings suggest that there is no definite DMR of *NNAT* in mice. With this background, we sought to identify the DMRs of mice *NNAT* via quantitative real-time PCR (q-PCR) and bisulfite sequencing PCR (BSP) analyses.

## Results and discussion

**Fig. 1:**
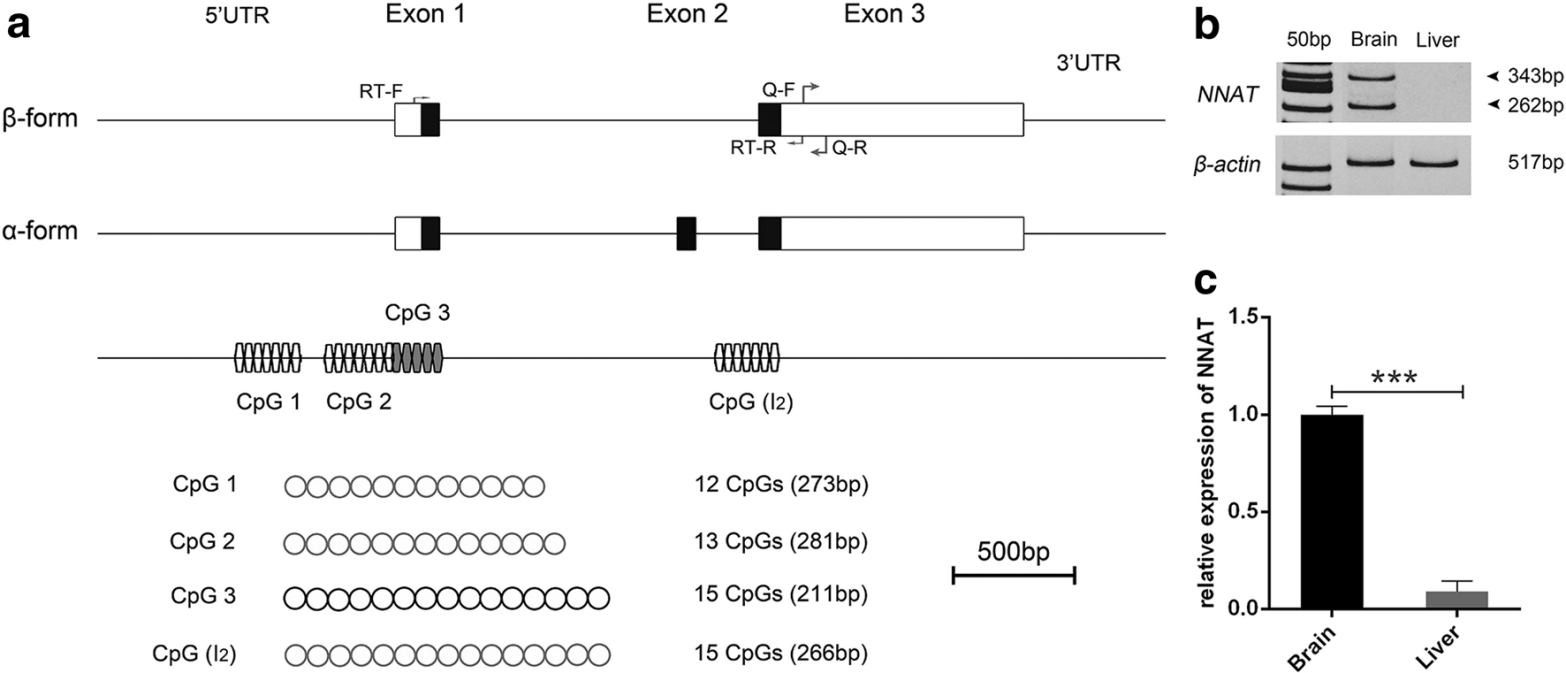
Gene structure and expression analysis of *NNAT* in mice. **a** Structure of the mice *NNAT* locus. *NNAT* features two alternatively spliced transcripts (α- and β-form). Protein coding regions are shown as *black*, *filled boxes*. *Circles* indicate CpG islands located in *NNAT* and analyzed via BSP. Q: Primers for q-PCR; RT: Primers for RT-PCR; **b** RT-PCR was used to detect the expression of two alternatively spliced isoforms in both brain and liver. **c** Gene expression of *NNAT* in brain and liver determined via q-PCR. Data are shown as Mean ± SEM (n = 6), *p < 0.05, **p < 0.01, ***p < 0.001

In this study, we proposed a model of two *NNAT* isoforms (α and β) using GenBank and Ensembl databases. Furthermore, putative DMRs of *NNAT* (CpG 1 and CpG 2, CpG 3, CpG I2) were identified via comparative sequencing analysis and methyprimer (Fig. 1a). The results of q-PCR and RT-PCR demonstrated high expression of both of the transcripts (α and β) in the brain of mice, but not in their liver, which is consistent with the UniGene database (Fig. 1b, c). Previous studies have found two transcripts of *NNAT* that are widely expressed in most of tissues, including in livers and kidneys of cattle (both fetal and adult) and in 2-month-old pigs (Zaitoun and Khatib 2006; Cheng et al. 2007), suggesting different *NNAT* expression profiles between species.

**Fig. 2:**
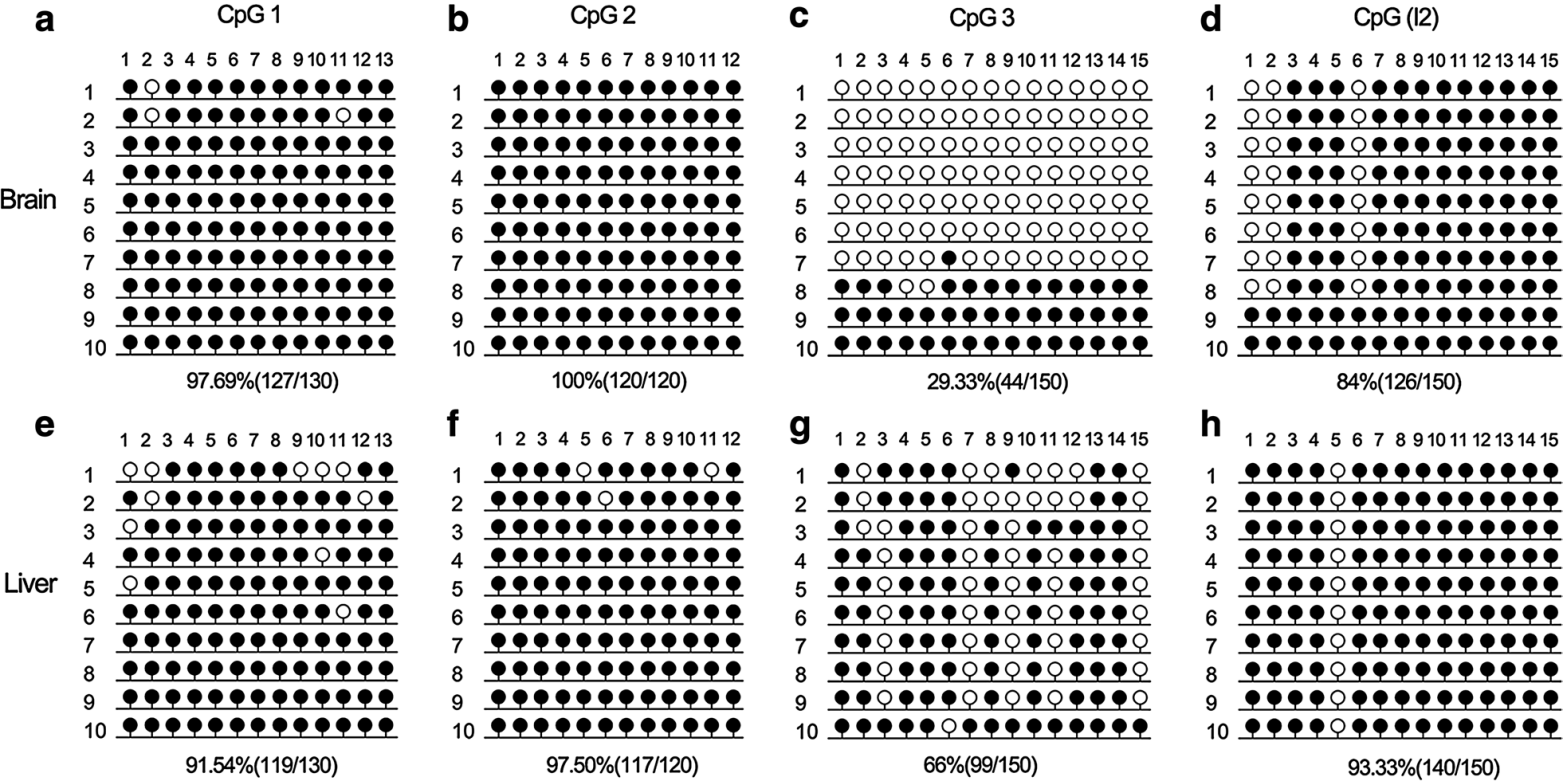
DMR identification of *NNAT* in mice. The methylation status was analyzed via BSP: The results are depicted for CpG 1 in brain **a** and liver **e**, for CpG 2 in brain **b** and liver **f**, for CpG 3 brain (**c**) and liver (**g**), and for CpG 3 in brain (**d**) and liver (**h**). *Open* and *closed circles* indicate unmethylated and methylated CpG sites, respectively. Numbers in parentheses represent the methylated CpG sites relative to all counted CpG sites

To identify the DMR of mice *NNAT*, methylation profiles of CpG 1, CpG 2, CpG 3, and CpG I2 were determined in both the brain and the liver using BSP. The results revealed hypermethylation of CpG1 (Fig. 2a vs. e), CpG2 (Fig. 2b vs. f), and CpG I2 (Fig. 2d vs. h) in both brain and liver. However, we found tissue-specific methylation patterns of CpG3 in the brain (29.33%) and liver (66%) (Fig. 2c vs. g). These results point towards the methylation status of CpG3 accounting for the expression difference of *NNAT* in liver vs. brain. In addition, tissue-specific methylation patterns revealed CpG3 (located on exon 1) to be a putative DMR of the *NNAT* in mice (Fig. 2c).

**Fig. 3:**
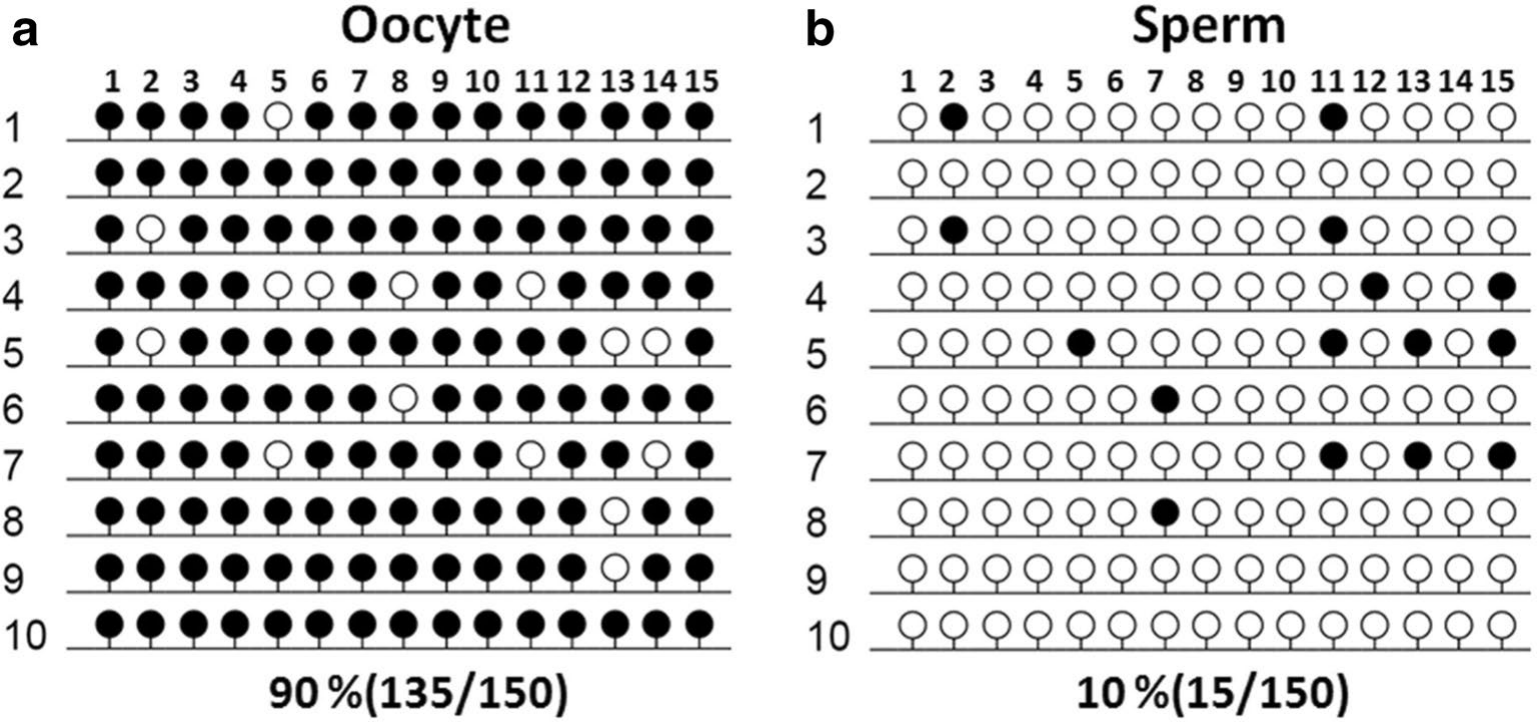
Gamete-specific methylation status of CpG 3. The methylation status of CpG 3 was analyzed via BSP for MII oocyte (**a**) and sperm (**b**). *Open* and *closed circles* indicate unmethylated and methylated CpG sites, respectively. Numbers in parentheses represent the methylated CpG sites relative to all counted CpG sites

Generally, DNA methylation patterns of imprinted genes were established in both sperm and oocyte, which were reprogrammed subsequent after fertilization (Seisenberger et al. 2013). To identify the gamete-specific methylation pattern of CpG3 in mice *NNAT*, the DNA methylation profile of CpG3 in sperm and oocyte was determined via BSP. The results reveal hypomethylation in sperm (10%) and hypermethylation in MII oocytes (90%) (Fig. 3). In summary, the gamete-specific methylation patterns suggest that the imprinting marks of *NNAT* are established by a sex-specific mechanism, confirming CpG3 as the DMR of *NNAT.*

## Conclusions

Here, we studied the expression patterns of two isoforms of mice *NNAT* (α and β), and identified the CpG3 of mice *NNAT* (located on exon 1) as putative DMR.The data revealed hypermethylation of exon 1 to be associated with the silencing of mice *NNAT*, suggesting the *NNAT* gene transcriptional status to be correlated with the methylation status of exon 1, while being independent of both intron and 5′ UTR.

## Methods

### Tissue samples

The brain and liver were collected from two-month old ICR mice and immediately stored in liquid nitrogen until further use. Spermatozoa and MII oocytes were harvested using previously published protocols (Chen et al. 2014).

### RT-PCR and quantitative real-time PCR (q-PCR)

Total RNA from liver and brain (n = 5) was isolated with TRNzol-A+ reagent (TIANGEN, Beijing, China) according to manufacturer’s instructions. The cDNA was synthesized with DNAse I (Fermentas, Shanghai, China) treated total RNA via the BioRTcDNA First Stand Synthesis Kit (Bioer Technology, Hangzhou, China).

The BioEasy SYBR Green I Real Time PCR Kit (Bioer Technology, Hangzhou, China) was used to perform q-PCR, using the BIO-RAD Iq5 Multicolor Real-Time PCR Detection System. The reaction conditions were as follows: 95 °C for 3 min, followed by 40 cycles of 95 °C for 10 s for DNA denaturation, 60 °C for 15 s for primer annealing, and 72 °C for 30 s for extension. Relative gene expression normalized to *β*-*actin* was determined via the 2^−ΔΔCT^ formula. All experiments on gene expression were performed in triplicate. The gene expression data was presented as mean ± SEM. Primers used for q-PCR and RT-PCR are listed in Table 1.

**Table 1:** Primers for q-PCR and RT-PCR

Gene	Primer sequence (5′ → 3′)	T_ann_ (°C)	Length (bp)
*NNAT*–RT	F:GCTCATCATCGGCTGGTACA	60	343
	R:CTTGGCAAGTGCTCCTCTGA		262
β-*actin*-RT	F:ATATCGCTGCGCTGGTCGTC	60	517
	R:AGGATGGCGTGAGGGAGAGC		
*NNAT*-q	F:GTCCCCTGTGTTCCCTCGTC	60	81
	R:TGTCGGTGCTGCTTTTCTGG		
*Actin*-q	F:GGCACCACACYTTCTACAATG	60	133
	R:GGGGTGTTGAAGGTCTCAAAC		

*T*_*ann*_ the annealing temperature, *RT* the primers for RT-PCR, *Q* the primers for q-PCR

### Bisulfite sequencing PCR

Bisulfite sequencing PCR (BSP) was performed to determine methylation of *NNAT* CpG islands. The CpGenome TM Turbo Bisulfite Modification Kit (Millipore, Jaffrey, NH, USA) was used for bisulfate treatment of genomic DNA of both liver and brain according to the manufacturer’s instructions. Bisulfite-treatment of the DNA of sperms (n = 1 × 10^3^) and matured oocytes (n = 100) was done according to the instructions of the EZ DNA Methylation-Direct™ Kit (Zymo Research,CA). Nested PCR was used to amplify CpG islands, followed by T-vector cloning (positive clones, n = 10) and subsequent sequencing analysis. The BSP primers are listed in Table 2.

**Table 2:** Primers for BSP

Gene	Primer sequence (5′ → 3′)	T_ann_ (°C)	Length (bp)
CpG1-BSP	O-F:TTTTGTGTTTTAGTTGTATAGCGAA	55	504
	O-R:AATACAAACCTCTTAATTCGACACA		
	I-F: TAGAGGTTCGTATTTGTTTCGTAG	55	275
	I-R:TTTTTCTACATTCCTACTAATCCGT		
CpG2-BSP	O-F:GAGCGGGAATTAATAGTTAGAAAAG	55	526
	O-R:ACTAATCTCGAAATCCGCTACTAAA		
	I-F:GTATGTAGAATTTGTAGGTTTGGG	54	282
	I-R:CTCTTACCACCTAAATACGCATAC		
CpG3-BSP	O-F:GGTAGAGTAGAATTTTTTGGA	58	590
	O-R:CACCCCTAAATCTTTATTCCC		
	I-F:TTTAGGTGGTAAGAGGGTATTTAAGGTA	60	211
	I-R:AATACATACTCACCTACAACA		
I2-BSP	O-F:TTTGGAATGTTGTATTTATTGGGTAGGA	55	558
	O-R:CCCCTCACTAACCTTAACAAATACTCCTC		
	I-F:TAGTTGTTTTGATTGGTGGATAAGT	56	266
	I-R:AACTCGCTACCTACGCTCCC		

*T*_*ann*_ the annealing temperature, *O* outside primers of the BSP, *I* inside primers of the BSP

### Statistical analysis

Data were analyzed using student’s *t*-tests via SPSS 22.0 software (SPSS Inc., Chicago, IL, USA) and a *p* < 0.05 was considered as statistically significant. DNAman and online software tools Methprimer (http://www.urogene.org/methprimer/) and Bio Analyzer (http://biq-analyzer.bioinf.mpi-inf.mpg.de/tools/MethlationDiagrams/index.php) were used for the methylation analysis.

## Abbreviations

*NNAT*: neuronatin
PCR: polymerase chain reaction
qPCR: quantitative real-time PCR
BSP: bisulfite sequencing PCR
DMR: differentially methylated region
RT-PCR: reverse transcription PCR

## References

[CR1] Bonin CA, Lewallen EA, Baheti S, Bradley EW, Stuart MJ, Berry DJ (2016). Identification of differentially methylated regions in new genes associated with knee osteoarthritis. Gene.

[CR2] Chen X, Wang T, Lv Q, Wang A, Ouyang H, Li Z (2014). DNA methylation-mediated silencing of neuronatin (NNAT) in pig parthenogenetic fetuses. Gene.

[CR3] Cheng H-C, Zhang F-W, Deng C-Y, Jiang C-D, Xiong Y-Z, Li F-E (2007). NNAT and DIRAS3 genes are paternally expressed in pigs. Genetics Selection Evolution.

[CR4] Das R, Lee YK, Strogantsev R, Jin S, Lim YC, Ng PY (2013). DNMT1 and AIM1 Imprinting in human placenta revealed through a genome-wide screen for allele-specific DNA methylation. BMC Genomics 14685.

[CR5] Duan F, Chen X, Yuan L, Song Y, Wang A, Lv Q (2015). Conservation of imprinting of Neuronatin (Nnat) in rabbits. Springerplus..

[CR6] Gu T, Su X, Zhao S, Li C (2014). Methylation differences of the neuronatin gene promoter region in liver between normal and cloned pigs. Anim Genet.

[CR7] Hubertus J, Zitzmann F, Trippel F, Muller-Hocker J, Stehr M, von Schweinitz D (2013). Selective methylation of CpGs at regulatory binding sites controls NNAT expression in Wilms tumors. PLoS ONE.

[CR8] Kuerbitz SJ, Pahys J, Wilson A, Compitello N, Gray TA (2002). Hypermethylation of the imprinted NNAT locus occurs frequently in pediatric acute leukemia. Carcinogenesis.

[CR9] Li X, Hamano K, Qian XQ, Funauchi K, Furudate M, Minato Y (1999). Oocyte activation and parthenogenetic development of bovine oocytes following intracytoplasmic sperm injection. Zygote.

[CR10] Schulz R, McCole RB, Woodfine K, Wood AJ, Chahal M, Monk D (2009). Transcript- and tissue-specific imprinting of a tumour suppressor gene. Hum Mol Genet.

[CR11] Seisenberger S, Peat JR, Reik W (2013). Conceptual links between DNA methylation reprogramming in the early embryo and primordial germ cells. Curr Opinion Biol.

[CR12] Sowpati DT, Thiagarajan D, Sharma S, Sultana H, John R, Surani A (2008). An intronic DNA sequence within the mouse Neuronatin gene exhibits biochemical characteristics of an ICR and acts as a transcriptional activator in Drosophila. Mech Dev.

[CR13] Surani MA (1998). Imprinting and the initiation of gene silencing in the germ line. Cell.

[CR14] Ushijima T (2005). Detection and interpretation of altered methylation patterns in cancer cells. Nat Rev Cancer.

[CR15] Zaitoun I, Khatib H (2006). Assessment of genomic imprinting of SLC38A4, NNAT, NAP1L5, and H19 in cattle. BMC Genet.

